# A numerical study on heat transfer performances of horizontal ground heat exchangers in ground-source heat pumps

**DOI:** 10.1371/journal.pone.0250583

**Published:** 2021-05-19

**Authors:** Hang Zou, Peng Pei, Chen Wang, Dingyi Hao

**Affiliations:** 1 College of Mining, Guizhou University, Guiyang, Guizhou; 2 Key Laboratory of Deep Coal Resource Mining, Ministry of Education, China University of Mining and Technology, Xuzhou, China; Tongji University, CHINA

## Abstract

Horizontal ground heat exchangers (HGHEs) have advantages such as convenient construction and low cost; however, their application and popularization are restricted owing to traditional linear HGHEs occupying large space and presenting low total heat transfer capacity. Spiral-coil and slinky-coil HGHEs have been proposed, but currently a comprehensive comparison and evaluation for these types of HGHEs are still needed. In this study, a three-dimensional heat transfer model of the three types of HGHEs for ground source heat pumps (GSHPs) was established. Based on the simulation results, the long-term heat transfer performances were investigated, including the temperature field of surrounding energy-storage soils, outlet working fluid temperature, coefficient of performance (COP) of units, and surplus temperature of the energy-storage soils. A new concept named heat transfer capacity per heat-affected area was proposed in this paper. It is found that the spiral-coil HGHEs have the best performances in terms of working-fluid outlet temperature, unit COP, total heat transfer capacity, heat transfer rate heat-affected area. The linear HGHEs shows the best performances in terms of mitigating heat imbalance risk and heat transfer rate per length. The results provide a reliable basis for selection of HGHE types in engineering practice and improvement guide in the future.

## 1 Introduction

Ground-source heat pumps (GSHPs) have been widely used to utilize shallow geothermal energy. As a technology for renewable energy utilisation, the GSHP presents various advantages such as economical and efficient energy utilisation, no pollution, low operation cost, unrestricted by geological conditions. It is considered as a green energy technology of tremendous potential for building energy supply [1–3].

**Fig 1:**
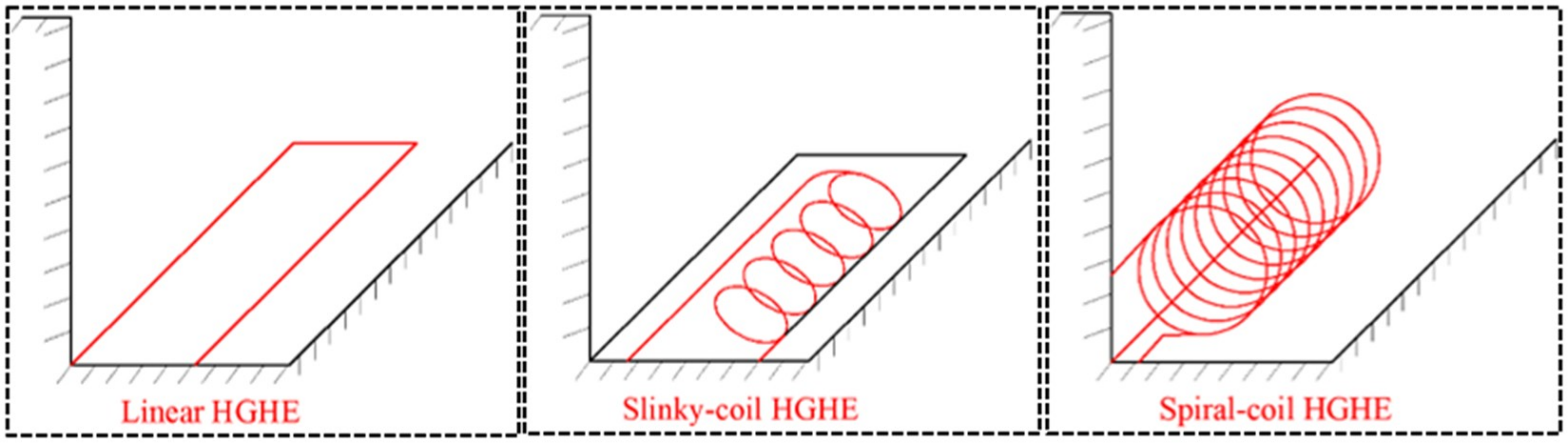
Different types of HGHEs.

As the main equipment in the system for heat transfer, the ground heat exchanger transfers heat between fluids in the tube and surrounding soils [4]. Therefore, the heat transfer performance of the ground heat exchangers is always of concern and has been explored by many scholars [5, 6]. Traditionally, horizontal ground heat exchangers (HGHEs) are linearly buried. In recent years, some new patterns of HGHEs (Fig 1) have appeared to improve the heat transfer performance, and they are popular due to some superiorities compared with the traditional ones [7]. Since the Slinky-coil is arrayed horizontally, it is easier to install. However, thermal interface among coils decreases heat transfer. The spiral-coil allows the tube contact the soil sufficiently and reduces thermal interface among coils, improving its efficient performance [7].

Engineers and researchers have conducted extensive research into the optimisation design of new patterns and structures. For example, Hikari et al. [8, 9] conducted numerical simulation on the optimisation design of slinky-coil HGHEs. The result showed that the simulated result agrees with the test result, verifying that the numerical model can be employed to simulate and investigate the heat transfer problem of a slinky-coil HGHE and can be used for their optimisation. Kim et al. [10, 11] conducted experimental and numerical studies to investigate the performances of spiral coils according to centre distances variation, and found that centre distance does not affect the thermal performance if the pitch exceeds 0.6 m. Li et al. [12] established a numerical model for determining the structural dimension of a spiral heat exchanger, which can be used to calculate the surface temperature of the spiral heat exchanger and provide a theoretical basis for its actual dimension design. Jeon et al. [13, 14] provided an evaluation index (*i*.*e*. load sharing ratio) for performances of spiral coil HGHEs, which can be used to determine the heat exchange rate of the spiral coils. Moreover, they constructed a three-dimensional (3D) numerical model for HGHEs to explore the factors influencing the load sharing ratio through numerical simulation. The research result indicated that the main factors influencing the load sharing ratio include the radius and the centre distance of spiral coils. When the coils feature a large ratio of the radius to centre distance, the load sharing ratio rises, which can be applied to predict the increase in ground temperature.

However, the main drawback of HGHEs is their relative lower total heat transfer capacity compared with that of vertical GHEs. Astanian et al. [15] investigated thermogravitational energy transport of variable viscosity liquid in a passive cooling system with porous insertion. The concept might be borrowed to improve the performance of heat carrying fluid of HGHEs. Some other advanced thermal storage media and technologies might be coupled with the HGHE to enhance its storage capacity, including multi-layer phase change materials which has been investigated to use in tubular heat exchanger [16], partially filled copper foam with Cu/GO nano-additives that have used in circular latent heat storage system [17], nano-encapsulated phase change particles and materials that might be used as energy carrying fluid in the HGHEs [18, 19]. Also, thermal capacity of backfill materials of HGHE ditches might be improved by mixing with phase change materials [20]. It is expected that these high heat capacity materials could increase the efficiency performance of the HGHEs.

In previous studies, there are no essential quantitative criteria on the advantage of different types of HGHEs; moreover, in practical engineering, the layout of HGHEs is restricted by the available area and therefore any type of HGHEs that can exchange more heat per available area, shows stronger applicability. Aiming at the aforementioned problem, by establishing the heat transfer models slinky-coil, spiral-coil, and linear HGHEs, their long-term heat transfer performances were analysed and compared, including total heat transfer capacity, heat transfer rate per length, heat transfer rate heat-affected area, working-fluid outlet temperature, unit COP and risk of heat imbalance. The study provides a reliable basis for selection of an appropriate type of HGHEs in engineering practice.

## 2 Theories and governing equations

Soil consists of solid grains, moisture and air. Water content of soils impacts the thermophysical properties and heat transfer between soil and heat exchanger, so it is taken as one of main factors influencing the heat transfer performance of ground heat exchangers. Owing to HGHEs being buried at shallow depths, the heat exchanger pipes are usually in an unsaturated zone of water in soils, whose heat exchange is a complex thermodynamic process coupling heat transfer with water migration under the synergistic effect of the temperature gradient and humidity gradient [21].

### 2.1 Hydraulic characteristics of soils

van Genuchten proposed the VG model in 1980 [22] to quantify the relationship between the water distribution and soil suction within the unsaturated zone above the water table, called the water retention curve of soils:

$$\theta =\theta_{r}+\frac{\theta_{s}-\theta_{r}}{{\left[1+{\left(ah\right)}^{n}\right]}^{m}}$$ (1)

where, *θ*, *θ*_*r*_, *θ*_*s*_, and *h* represent volumetric water content (cm^3^/cm^3^), retention rate (cm^3^/cm^3^) of water, saturated water content (cm^3^/cm^3^) and negative pressure (cmH_2_O), respectively; *a* is a scale parameter inversely proportional to mean pore diameter (cm^-1^); *n* and *m* separately denote the shape parameters of the characteristic curve of soil water (where, *m* = 1–1/*n*).

The above equation shows that the water content *θ* of unsaturated soils at different depths varies with the capillary suction *h*, which is related to the depth of the groundwater and the distribution of capillary water in such soils.

Soils are a multiphase system [4, 23]. To simplify the model to perform investigation and analysis, it is thought that soil consists of water, air, and soil skeleton (matrix), whose component contents are separately expressed as follows:

$$\text{Soil}\;\chi_{s}=1-\eta$$ (2)

$$\text{Water}\;\chi_{w}=\theta$$ (3)

$$\text{Air}\;\chi_{air}=\eta –\theta$$ (4)

where, *η* represents the soil porosity. Thus, based on different volume fractions of various components, the effective thermal conductivity *k*_*eff*_ and effective volumetric heat capacity (*ρC*_*p*_)_*eff*_ of the soil matrix are expressed as follows [24, 25]:

$$K_{eff}=\overset{3}{\underset{1}{\sum }}\chi_{i}k_{i}$$ (5)

$${\left(\rho C_{p}\right)}_{eff}=\overset{3}{\underset{1}{\sum }}\chi_{i}\rho_{i}c_{i}$$ (6)

Assuming that the porosity *η* of specific soils is fixed, the difference of the water content of soils is considered as the main reason for the difference of thermophysical properties of soils. Within a certain range, the water content of soils is positively correlated with the soil depth and thus the thermophysical parameters of soils at different depths are not constants but variables.

The water in soils flows under the effect of the potential difference: the total soil water potential *ψ* is composed of the gravity potential *ψ*_*g*_, pressure potential *ψ*_*p*_, solute potential *ψ*_*m*_, and matric potential *ψ*_*θ*_ (also called capillary potential) [26, 27], that is,

$$\psi =\psi_{g}+\psi_{p}+\psi_{\theta }+\psi_{m}$$ (7)

Water flow in the saturated soils below the water table is described by Darcy’s law:

$$\nu =-\frac{\kappa }{\mu }\nabla \psi$$ (8)

$$\nabla \psi =\nabla \psi_{g}+\nabla \psi_{p}$$ (9)

where, *v*, *κ*, *μ*, and ▽*ψ* denote the Darcy’s velocity (m/s), the permeability coefficient (m^2^) of porous media, the dynamic viscosity (Pa∙s) of fluids and the difference of water potentials at any two points, respectively.

The unsaturated soils within the zone above the water table are described by applying Richards Equation; similarly, water flows from a position with a high potential to another position with a low potential:

$$\nu =-\frac{\kappa (\theta )}{\mu }\nabla \psi$$ (10)

$$\nabla \psi =\nabla \psi_{\theta }+\nabla \psi_{g}$$ (11)

where, *κ(θ)* represents the permeability coefficient which varies with the water volume fraction.

### 2.2 Heat transfer in soils

The energy conservation equation in the soil matrix is given by [28]:

$${\left(\rho C_{p}\right)}_{eff}\frac{\partial T}{\partial t}+\rho_{w}C_{p,w}v\nabla T+\nabla (-k_{eff}\nabla T)=Q_{wall}$$ (12)

where, *ρ*, *C*_*p*_, *T*, *t*, and *ρ*_*w*_ refer to the density (kg/m^3^) of porous media, the specific heat capacity (J/kg∙K) at constant pressure, the temperature (K), time (s), and the density of groundwater, respectively; *v*, *k*, and *Q*_*wall*_ denote the fluid velocity field (the velocity field in the saturated zone is coupled with Darcy’s law: that in the unsaturated zone with that in Richards equation) in the soil matrix, the coefficient of thermal conductivity (W/m∙K) and the heat transfer between the heat exchanger tube and their ambient environment (W/m^3^), respectively; *k*_*eff*_ and (*ρC*_*p*_)_*eff*_ refer to the effective thermal conductivity (W/m∙K) and effective volumetric heat capacity (J/kg∙K) of the soil matrix, respectively.

### 2.3 Heat transfer in HGHEs

The energy equation for the incompressible fluid flow in the HGHEs is given by [28]:

$$\rho_{f}AC_{p,f}\frac{\partial T_{f}}{\partial t}+\rho_{f}AC_{p,f}u\nabla T_{f}=\nabla Ak_{f}\nabla T_{f}+\frac{fD\rho_{f}A}{2d_{h}}{\left|u\right|}^{3}+Q_{wall}$$ (13)

where, *ρ*_*f*_, *A*, *C*_*p*,*f*,_ *T*_*f*_, *t*, and *u* stand for the fluid density, the cross-sectional area (m^2^) of the fluid flow, the specific heat capacity (J/kg∙K) at constant pressure of fluids in heat exchanger tubes, the temperature of circulated fluids, time (s) and the velocity (m/s) of the circulated fluids in ground heat exchangers, respectively; *k*_*f*_, *f*_*D*_, and *d*_*h*_ represent the coefficient of thermal conductivity (W/m∙K) of circulated fluids, Darcy’s friction coefficient and hydraulic mean diameter (mm).

Additionally, *Q*_*wall*_ is the heat transfer between the heat exchanger tube and their ambient environment, shown as follows:

$$Q_{wall}={\left(hZ\right)}_{eff}(T_{ext}-T)$$ (14)

where, (*hZ*)_*eff*_ refers to the effective value of the thermal conductivity, in which *h*, *Z*, and *T*_*ext*_ denote the equivalent convection heat transfer coefficient (W/m^2^∙K) of the pipe wall, the perimeter (m) of the pipe wall, and the temperature (K) of the porous media outside the pipe wall, respectively.

## 3 Numerical simulation

### 3.1 Numerical model

Based on the hydraulic characteristics of soils, the distribution of water from the surrounding soils of HGHEs is identified. The soil is assumed as loam soil. A 3D model for the heat transfer from a line source under different HGHEs is established to simulate the heat transfer performance. In the model, the HGHEs is buried 2 m deep and the water table is 6 m below the HGHEs. The soils around the HGHEs are unsaturated (Fig 1). The upper boundary of the model is set as a constant surface temperature of 20 °Cwithout water flux; the other 5 sides of the model have mass flux (water and air) and heat transfer.

The heat exchangers are lay in the horizontal trench of 2 m wide, 2 m deep and 30 m long. The horizontal projected area of the trench is defined as the actual available area for arranging the heat exchangers; and different types of HGHEs are discussed in terms of the same avaible area for layout. The simulation paratmers are summarized in Table 1.

**Table 1:** Simulation parameters.

Parameter	Value	Unit
Inner diameter of HGHEs	20	mm
Center distance of Spiral-coil HGHEs	0.4	m
Radius of Spiral-coil HGHEs	0.5	m
Center distance of Slinky-coil HGHEs	0.4	m
Radius of Slinky-coil HGHEs	0.5	m
Coefficient of thermal conductivity of the pipe wall	0.5	W.m^-1^.K^-1^
Axial length of spiral coils	30	m
Inlet temperature of working media	35	°C
Initial temperature of soils	15	°C
Long-term average surface temperature	20	°C

**Fig 2:**
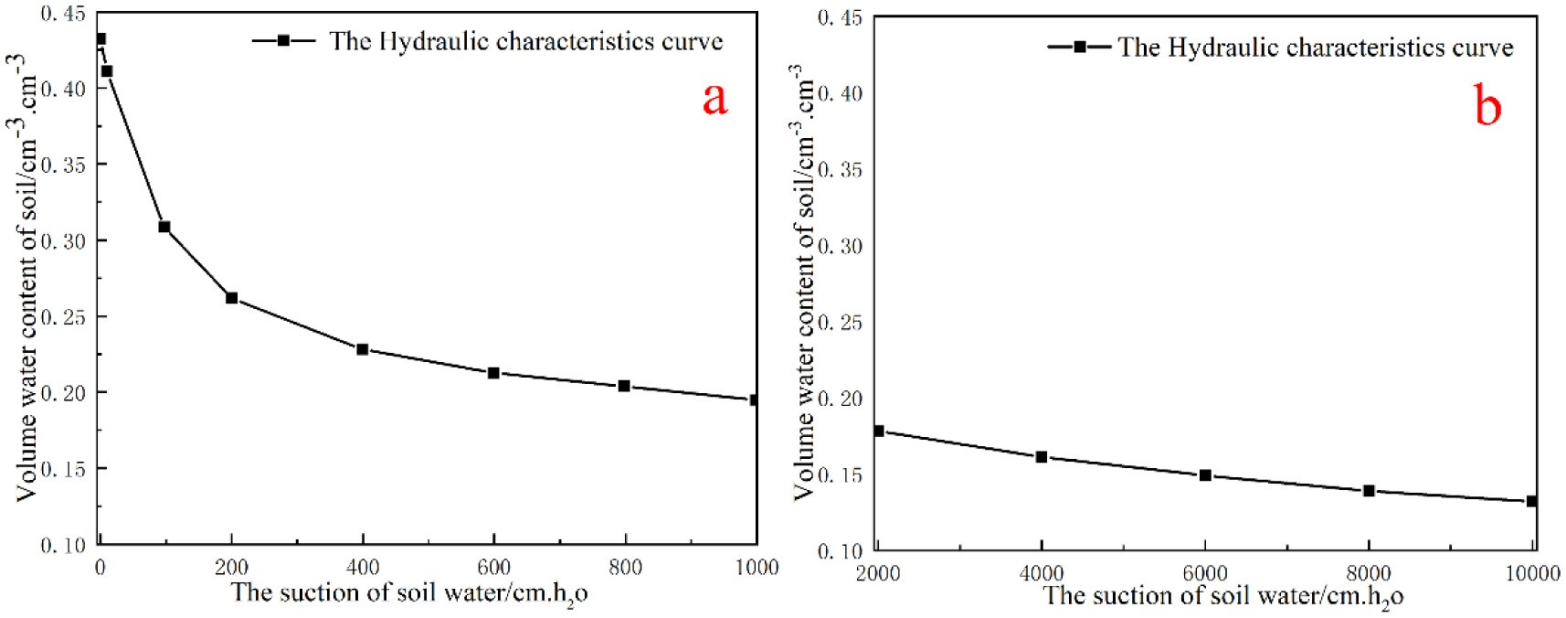
Retention curve of soil (a shows the low suction sector; and b shows the high suction section) [21].

The parameters of V-G model used in construct the water retention curve in this study are listed in Table 2 [29] and the retention curve of soil is shown in Fig 2.

**Table 2:** Hydraulic characteristic parameter of the soil.

Parameter	Symbol	Value
Saturated water content	*θs*	0.434
Residual water content	*θr*	0.052
Scale paramter	*a*	0.058 cm^-1^
Shape parameter	*n*	1.232

**Fig 3:**
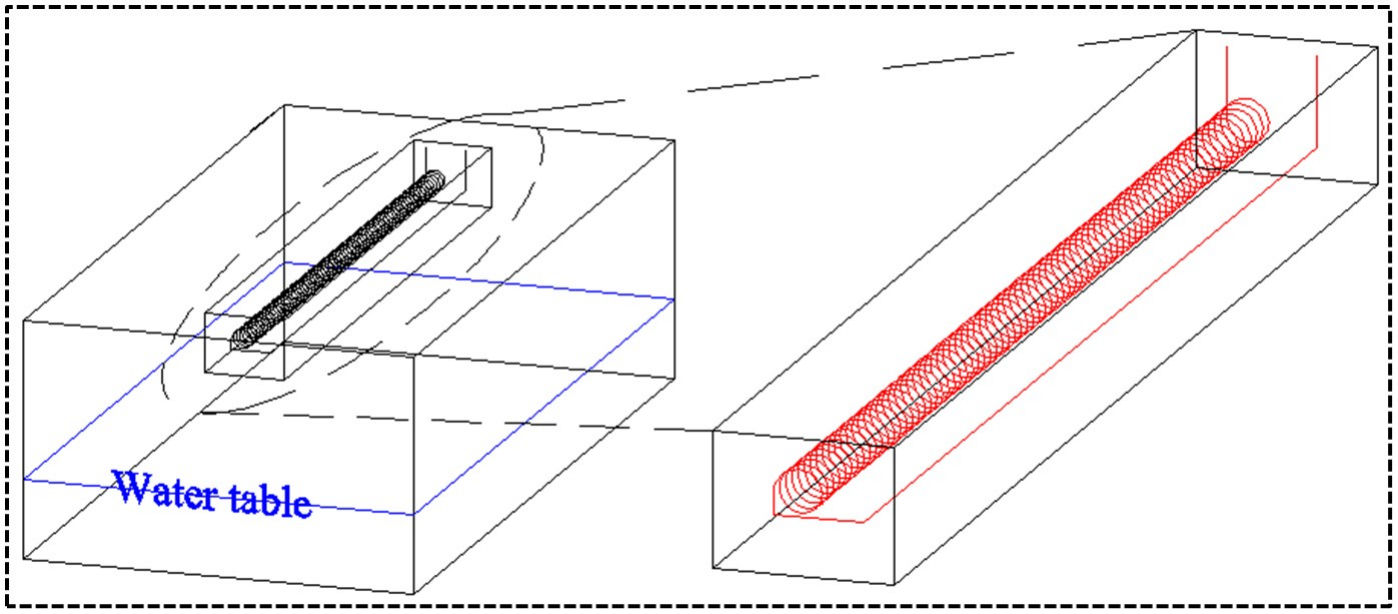
The energy-storage soil.

In order to describe the change trend of the temperature field around the HGHEs, a cuboid measuring 3 m × 3 m × 32 m is defined as the energy-storage soil body in the model. The excavated trench is located within the energy-storage soil, as shown in Fig 3.

### 3.2 Model meshing

**Fig 4:**
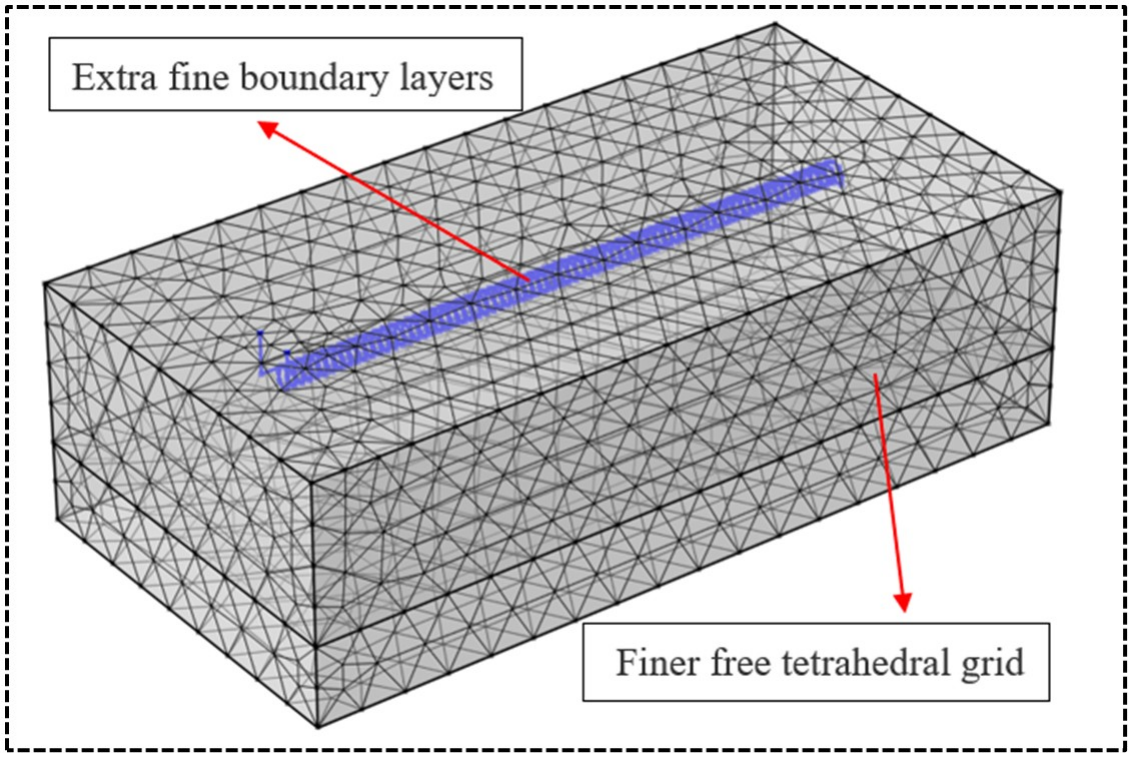
Meshing diagram.

In order to guarantee the accuracy of the simulation and demonstrate the grid-independence of the results, four meshing approaches were tested by the authors: I (24143 elements), II (29598 elements), III (40976 elements), and IV (86525 elements). Correspondingly, the calculated water outlet temperatures from the spiral HGHE are 303.68K, 303.77K, 303.83K and 303.84K respectively. It shows that Approaches I and II offers lower accuracy while Approaches III and IV both offer similar results and satisfy accurate requirements. Therefore, Approach III is selected. In this approach, the model is divided into two regimes to mesh (Fig 4): the horizontal tube which is meshed by extra fine boundary layers, and the soil which is meshed by finer free tetrahedral grid.

### 3.3 Conditions and assumptions

#### 3.3.1 Initial conditions

$$T(x,y,z)=T_{0}(T_{0}=15{}^{\circ}\text{C})$$ (15)

#### 3.3.2 Boundary conditions

1. Top boundary condition of the soil
  $$T(x,y,0)=T_{s}(T_{s}=18{}^{\circ}\text{C})$$ (16)
2. Bottom boundary condition of the soil
  $$T(x,y,-12)=T_{0}(T_{0}=15{}^{\circ}\text{C})$$ (17)
3. Remote boundary condition of the soil
  $$T(x,0,z)=T_{0}(T_{0}=15{}^{\circ}\text{C})$$ (18)
  $$T(x,-20,z)=T_{0}(T_{0}=15{}^{\circ}\text{C})$$ (19)
  $$T(0,y,z)=T_{0}(T_{0}=15{}^{\circ}\text{C})$$ (20)
  $$T(40,y,z)=T_{0}(T_{0}=15{}^{\circ}\text{C})$$ (21)

#### 3.3.3 Assumptions

1. The initial temperatures of HGHEs and soils are both 288.15K.
2. Influential of terrestrial heat flow to the bottom boundary is neglected.
3. The modelled area is bellow an indoor parking lot, therefore, only heat conduction exists between the top boundary of the soil and surface air, and convection is neglected.
4. Seepage of terrestrial water is neglected.

### 3.4 Model validation

**Fig 5:**
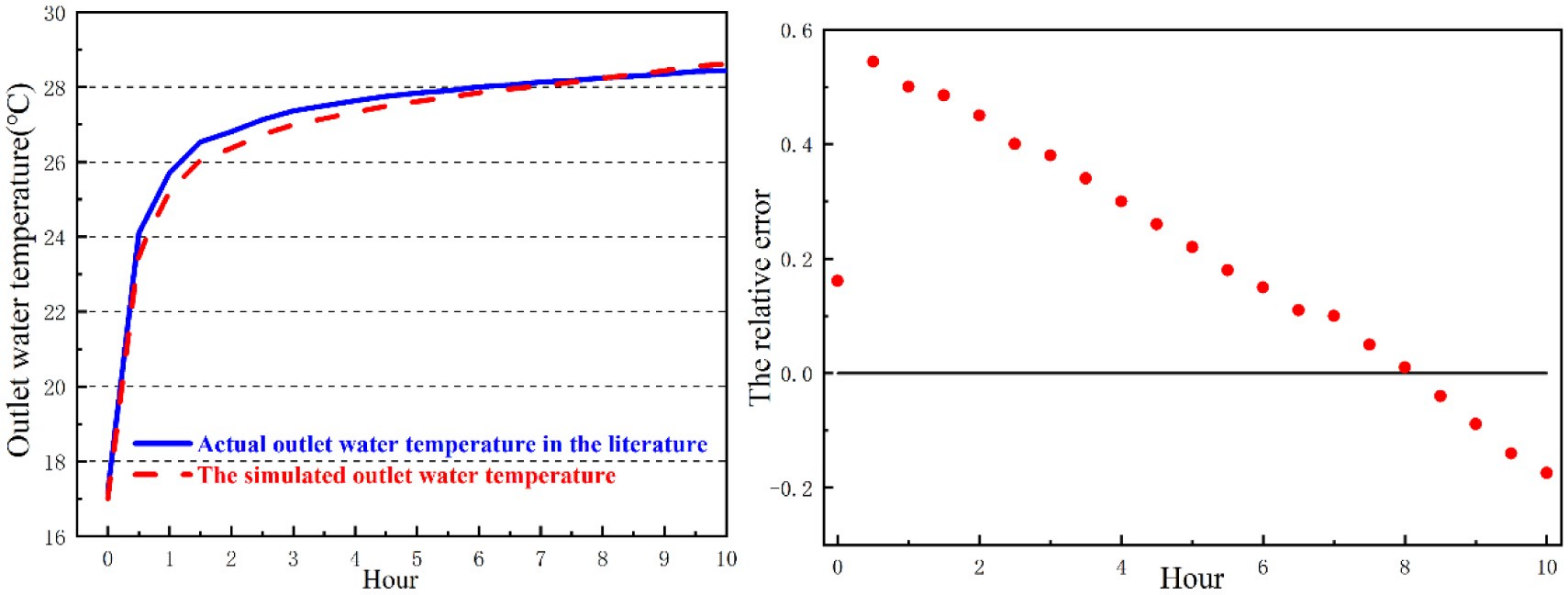
Comparison between the simulated outlet water temperature and the outlet water temperature from literature for verification.

To guanrantee the reliability of the model, it is necessary to verify the mathematical model before performing numerical simulation. Xu [7] simulated the ground heat transfer performance of spiral-coil HGHEs (Table 3) and validated the model by combining his findings with corresponding physical experiments. the simulation parameters from Xu [7] were used to verify the model built in the study;In the validation work, the outlet water temperature of a HGHE simulated by using the model built in this study was compared with that from Xu [7]. The results are as shown in Fig 5.

**Table 3:** Simulation and verification parameters.

Parameter	Value	Unit
Inner diameter of HGHEs	26	Mm
Thickness of the pipe wall	3	Mm
Depth of HGHEs	1.5	M
Diameter of spiral coils	0.3	M
Center distance of spiral coils	0.4	M
Axial length of spiral coils	15	M
Inlet water temperature of HGHEs	35	°C
Initial temperature of soils	17	°C

As shown in Fig 5, the change trend of the outlet water temperature of ground heat exchangers simuliated by the model in this study is similar to that shown in previous research of Xu [7], showing the acurancy and realiability of the model.

## 4 Simulation results

In order to quantify the extend of heat imbalance and subsequent impact to heat transfer under different types of HGHE more straightforwardly, an extreme situation was considered—the load was only in the cooling mode, which means heat was only injected into the ground but not extracted. The cooling season was assumed to be 90 days.

**Fig 6:**
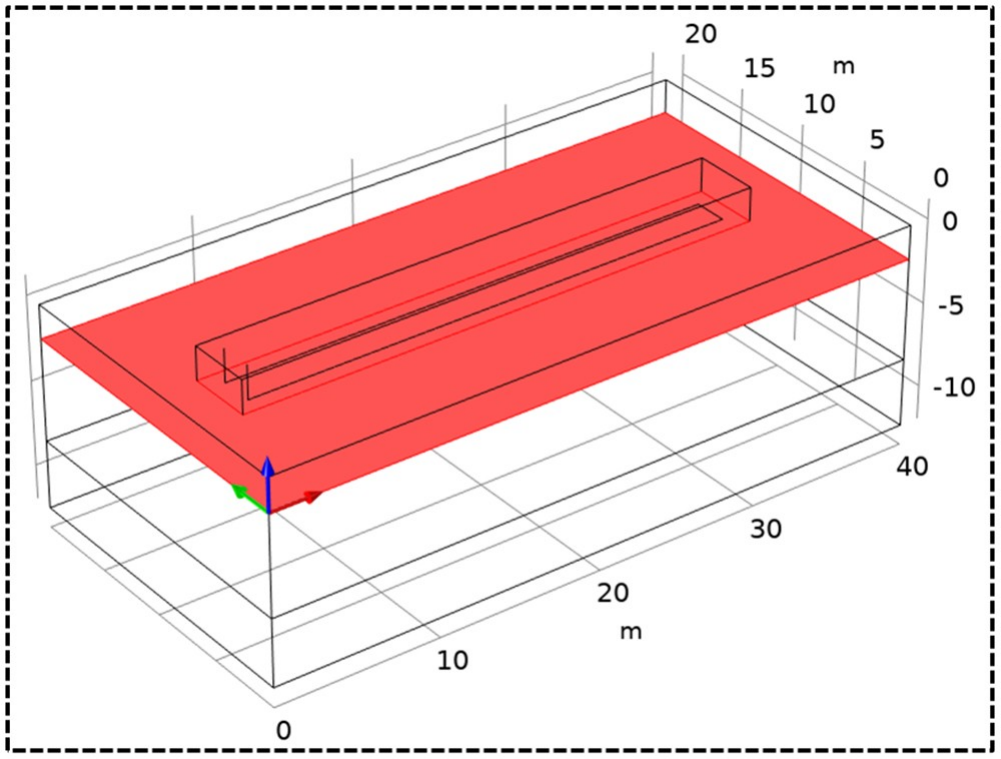
The X-Y section.

Fig 6 shows the dimension of the model. The *X*-*Y* section corresponds to the horizontal plane of a HGHE, with the cutting depth of the plane of 2 m.

As the high-temperature working media in HGHEs constantly transfer heat to soils, the soil temperature around the HGHEs increases. For the convenience of discussion and analysis, only the distribution of the temperature field on the *X*-*Y* section in the final cooling stage (the 90^th^ day) is shown in the result.

**Fig 7:**
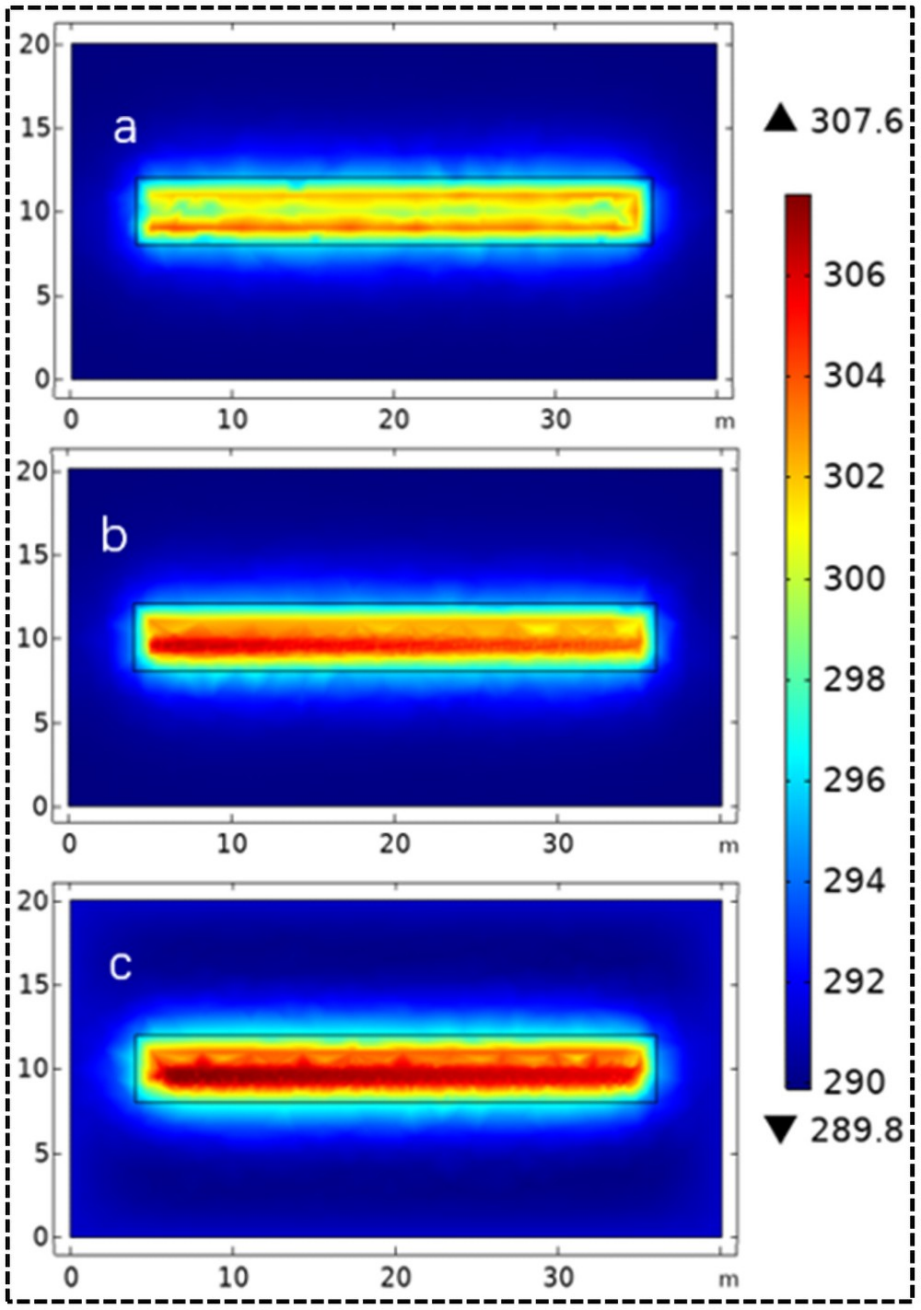
Temperature field in the final cooling stage: (a) linear HGHEs; (b) spiral-coil HGHEs; (c) slinky-coil HGHEs.

As shown in Fig 7, high-temperature zones appear in the vicinity of the HGHEs due to constant input of heat. It indicates that after cooling for 90 d, the soil temperature around the linear HGHEs rises to 304.4 K at most; the soil temperature around the spiral-coil HGHEs increases to 306.9 K and that in the vicinity of the slinky-coil HGHEs to 307.6 K, some 3.2 K higher than the soil temperature around linear HGHEs.

In addition, heat accumulation is significant in soils around slinky-coil HGHEs, where the temperature field is unevenly distributed. The heat transfer performance of the HGHEs is greatly affected by the temperature field. By contrast, the temperature field distribution of soils in the vicinity of linear HGHEs is relatively even.

## 5 Discussion and analysis

Based on the simulation results, various related parameters (including the distribution of the temperature field, thermal interference of water-supply and water-return sides of heat exchanger tubes, outlet water temperature of ground heat exchangers, coefficient of performance (COP) of units, economic benefit indices, and surplus temperature of energy-storage soils) are calculated and investigated. By doing so, it attempts to reveal the heat transfer performances of ground heat exchangers of different types of HGHEs and illustrate the pros and cons of each type. It provides a reliable basis for selection and installation of ground heat exchangers.

### 5.1 The temperature field in soils

As shown in Fig 7(a), given a fixed available area for installation of heat exchangers, the temperature field of linear HGHEs is better than the other two types as the temperature raised area is smaller; however, the total length of linear HGHEs is short and hence its heat transfer capacity is lower. By comparing Fig 7(b) and 7(c), the temperature field distribution of spiral-coil HGHEs is superior to that of slinky-coil HGHEs. The reason for this is that there is a short spacing between slinky-coil HGHEs which attribute to significant thermal interference between heat exchanger tubes. The soils around ground heat exchangers bear a large thermal load and the heat is hard to diffuse, thus leading to heat accumulation and a risk of system breakdown under long-term operation. The spacing between spiral coils is 0.4 m, which mitigates the thermal interference between heat exchangers to some extent and improves the comprehensive heat transfer performances of HGHEs.

### 5.2 Thermal interference in water-supply and working fluid-return sides of HGHEs

**Fig 8:**
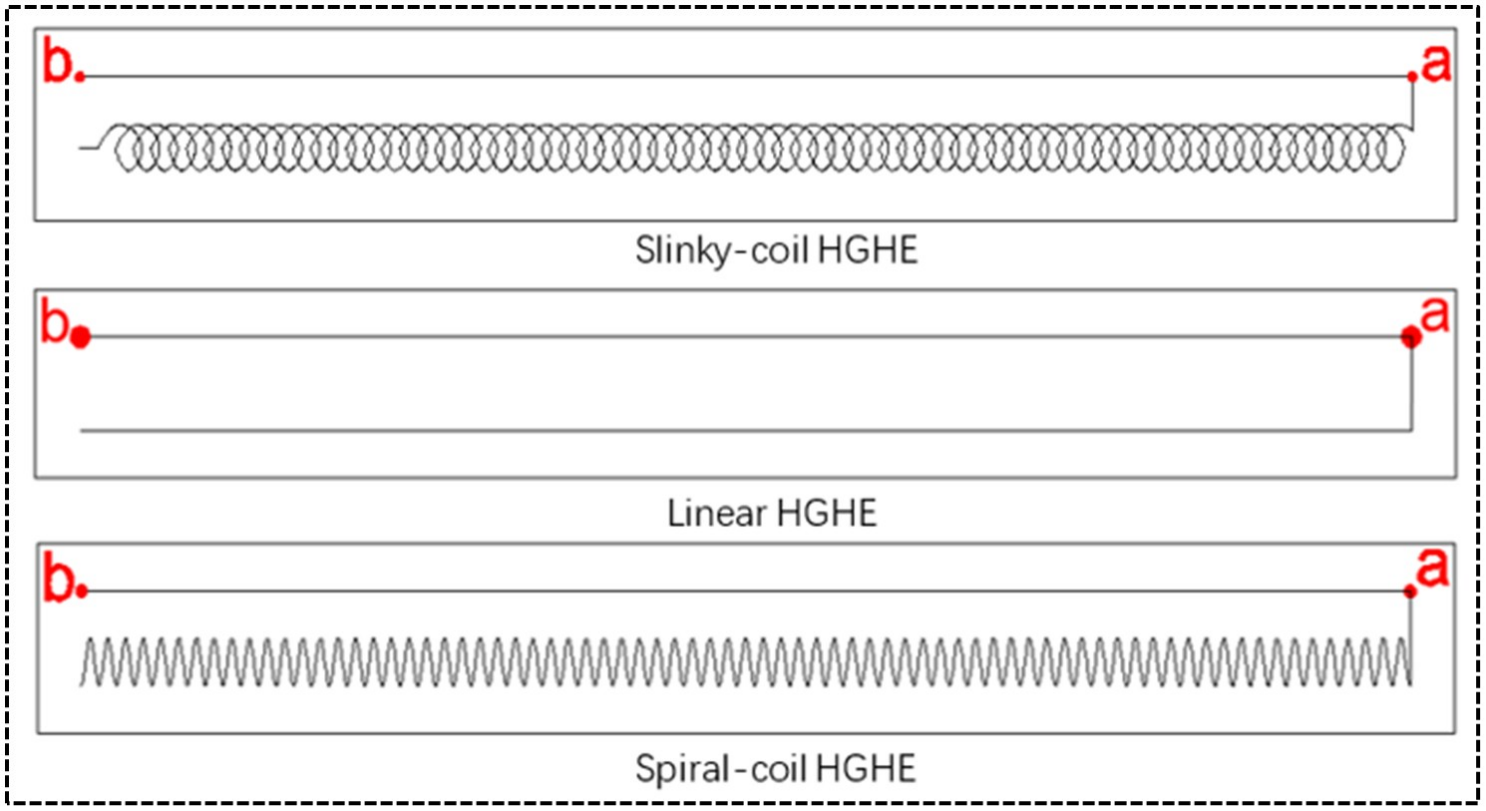
Nodes in the working fluid output end of different HGHE types.

**Fig 9:**
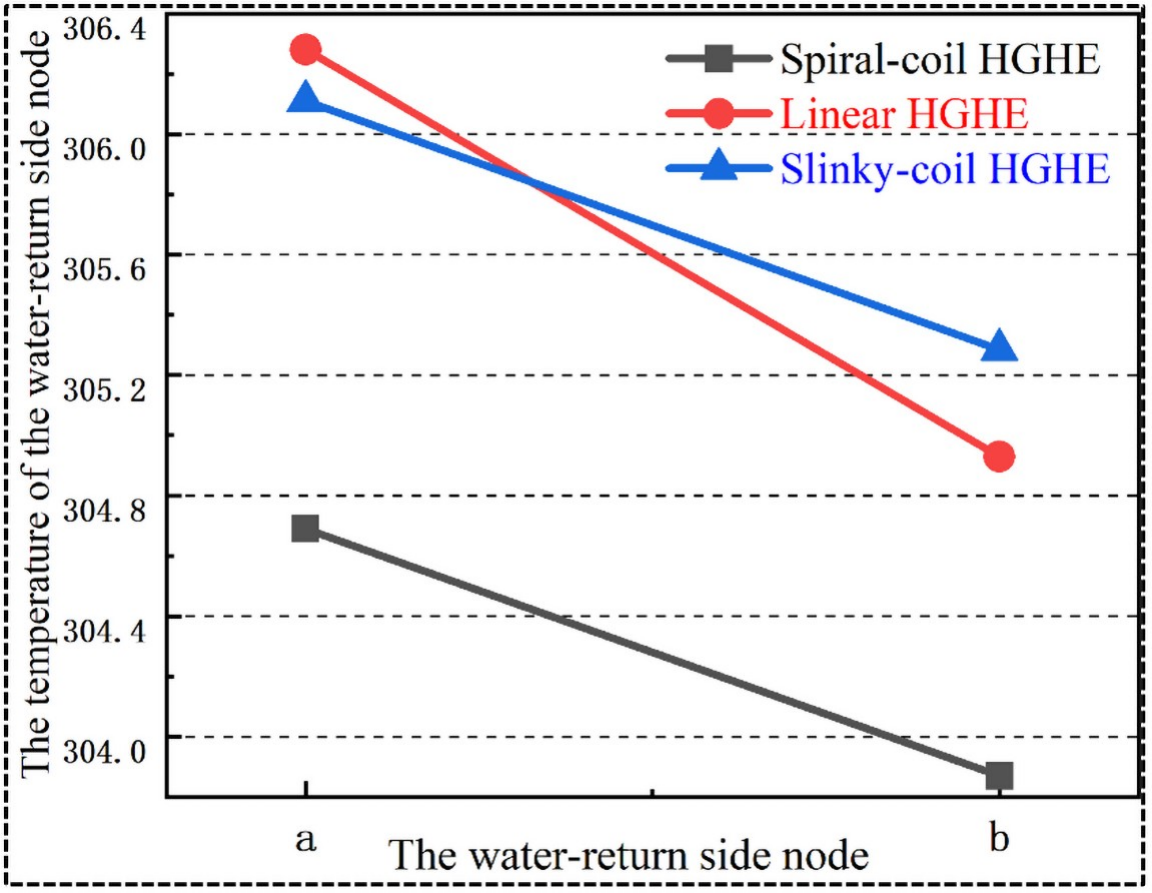
The changes in nodal temperature in the output end of HGHEs.

In practice, during construction, the working fluid input and output ends of the HGHEs are usually close to each other due to being constrained by the site area available for HGHEs, leading to thermal interference between the returning and input sections, and further affecting the heat exchange performances of units. To assess the extent of this thermal interference in different types of HGHEs, the corresponding nodes of HGHEs in the input and output ends of working fluid were selected (Fig 8) and the changes in temperature were analysed (Fig 9).

As shown in Figs 8 and 9, the returning working media flow through node *a* after adequate heat transfer with soils. In this case, in terms of the temperature of node *a*, the HGHEs are (in ascending order): spiral-coil HGHEs, slinky-coil HGHEs, and linear HGHEs.

Afterwards, the working media flow to node *b* which is the output end. In this condition, the temperature profile is changed and HGHEs in ascending order are: spiral-coil HGHEs, linear HGHEs, and slinky-coil HGHEs. Through analysis, it can be found that the return side of slinky-coil HGHEs is significantly affected by thermal interference between the inlet pipes. With the same length of return pipes, the heat transfer performance of slinky-coil HGHEs is inferior to that of linear HGHEs. The heat transfer performance of the returning section of spiral-coil HGHEs is weaker than that of linear HGHEs owing to the temperature difference between node *a* thereof and surrounding soils being relatively low. This is indicated by the slope of the curve corresponding to the spiral-coil HGHEs is lower than that of linear HGHEs in Fig 9.

### 5.3 Analysis of heat transfer

**Fig 10:**
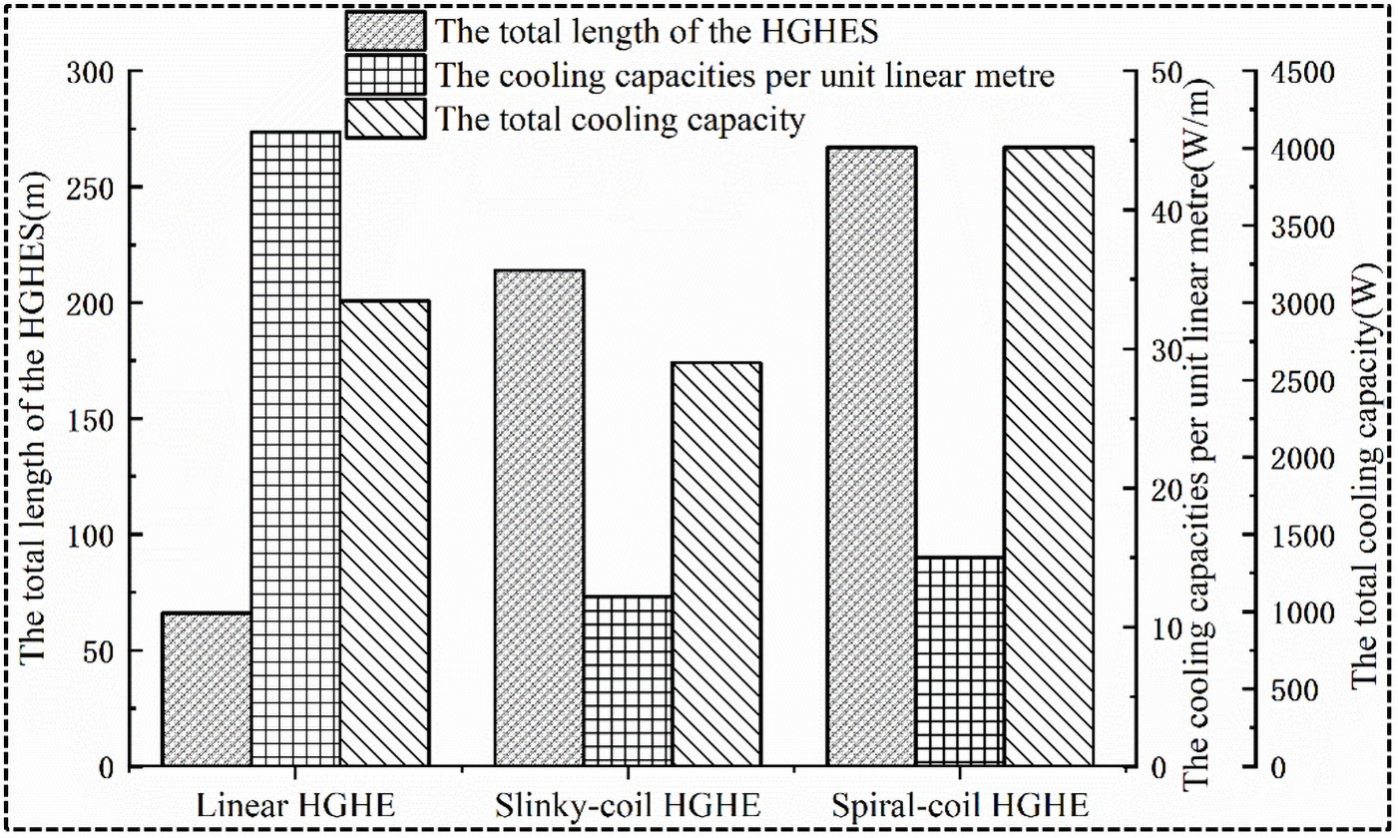
Comparison of heat transfer capacities of HGHEs.

In practice, the actual area for arranging HGHEs is generally restricted by the available space. Therefore, the contractors need to consider whether the total length of heat exchangers can satisfy the requirement for heat transfer capacity. As shown in Fig 10, within the same available area to arrange heat exchangers, the total length of linear HGHEs that can be installed can reach 66 m; while 214 and 267 m-long slinky-coil and spiral-coil HGHEs can be buried, respectively. The spiral-coil HGHEs presents the highest land utilisation ratio and hence the heat transfer area between heat exchangers and soils is large.

Owing to the finite thermal storage capacity of soils, the soils cannot accommodate additional heat when the heat input surpluses its storage capacity. Therefore, excessively increasing the total length of ground heat exchangers will actually sacrifice the heat transfer rate per length and leads to a waste of capital investment. The results show that the cooling capacities per linear metre of spiral-coil and slinky-coil HGHEs are 15 and 12.2 W/m, respectively; the heat transfer capacity per linear metre of linear HGHEs reaches 45.6 W/m at most, which is 274% larger than that of slinky-coil HGHEs. The linear HGHEs show the most significant unit economic benefit; however, since its total length is the shortest, it total cooling capacity is reduced.

For the slinky-coil HGHEs, its heat transfer rate per length is the lowest, as its working fluid return section is significantly influenced by the thermal interference. Thus, the slinky-coil HGHEs have the lowest total cooling capacity among the three types even though its total tube length is higher than that of the linear type.

The spiral-coil HGHs has the longest total length. At the same time, a single spiral coil transfers heat with the soils radically, but the spiral coils are lay along the axial direction, hence mitigating thermal interference among individual coils, and increasing its heat transfer per length higher than the slinky type. These effects give the spiral type the highest total heat transfer capacity or cooling capacity among the three types.

In engineering practice, the layout of ground heat exchangers is generally restricted by the availability of space, and it is expected to reach higher heat transfer capacity within a finite site area. In this study, two new evaluation indices: 1) the heat affected area, and 2) the cooling capacity per unit heat affected area are proposed. The benefits of the cooling capacity under different types of HGHEs are further compared. The projected area of the heat transfer affected zone of the HGHEs is defined as the heat affected area. The heat transfer capacity per heat-affected area is numerically expressed as the ratio of the total heat transfer power to the heat-affected area. A higher value of the heat transfer capacity per heat-affected area means greater the amount of exchanged heat within the same heat transfer area, and a higher land utilization efficiency.

**Fig 11:**
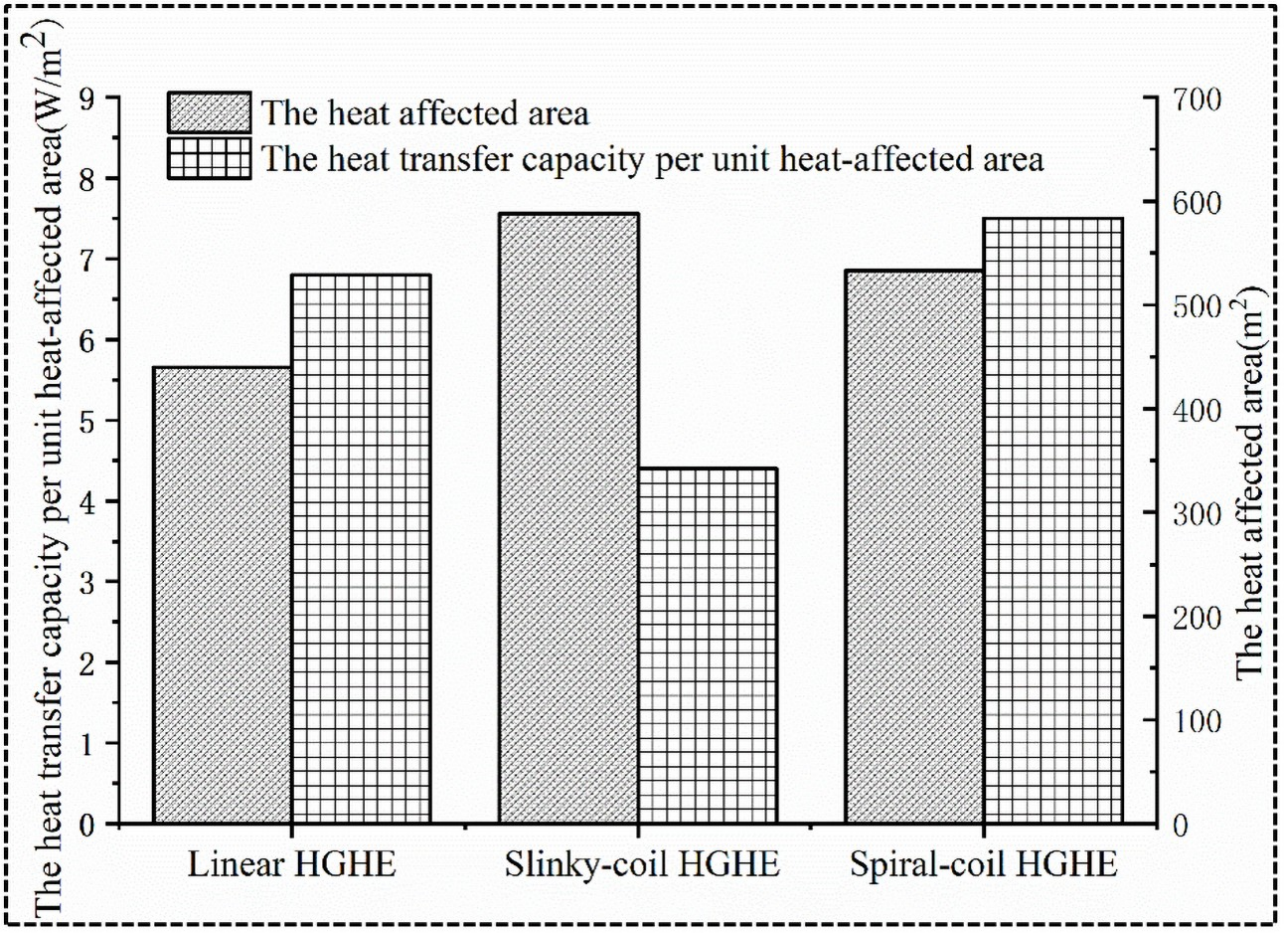
Comparison of heat transfer per heat-affected area.

Through analysis of the data in Figs 10 and 11, it can be found that slinky-coil HGHEs present the lowest cooling capacity per heat-affected area. The total cooling capacity of spiral-coil HGHEs is 955 W higher than that of linear HGHEs while their heat-affected area is only 93 m^2^ greater than that of the linear HGHEs. Among HGHEs with three HGHE types, the spiral-coil HGHEs exhibit the largest cooling capacity per heat-affected area, thus showing an optimal economic performance.

### 5.4 Outlet temperature of working fluid from heat exchanger

**Fig 12:**
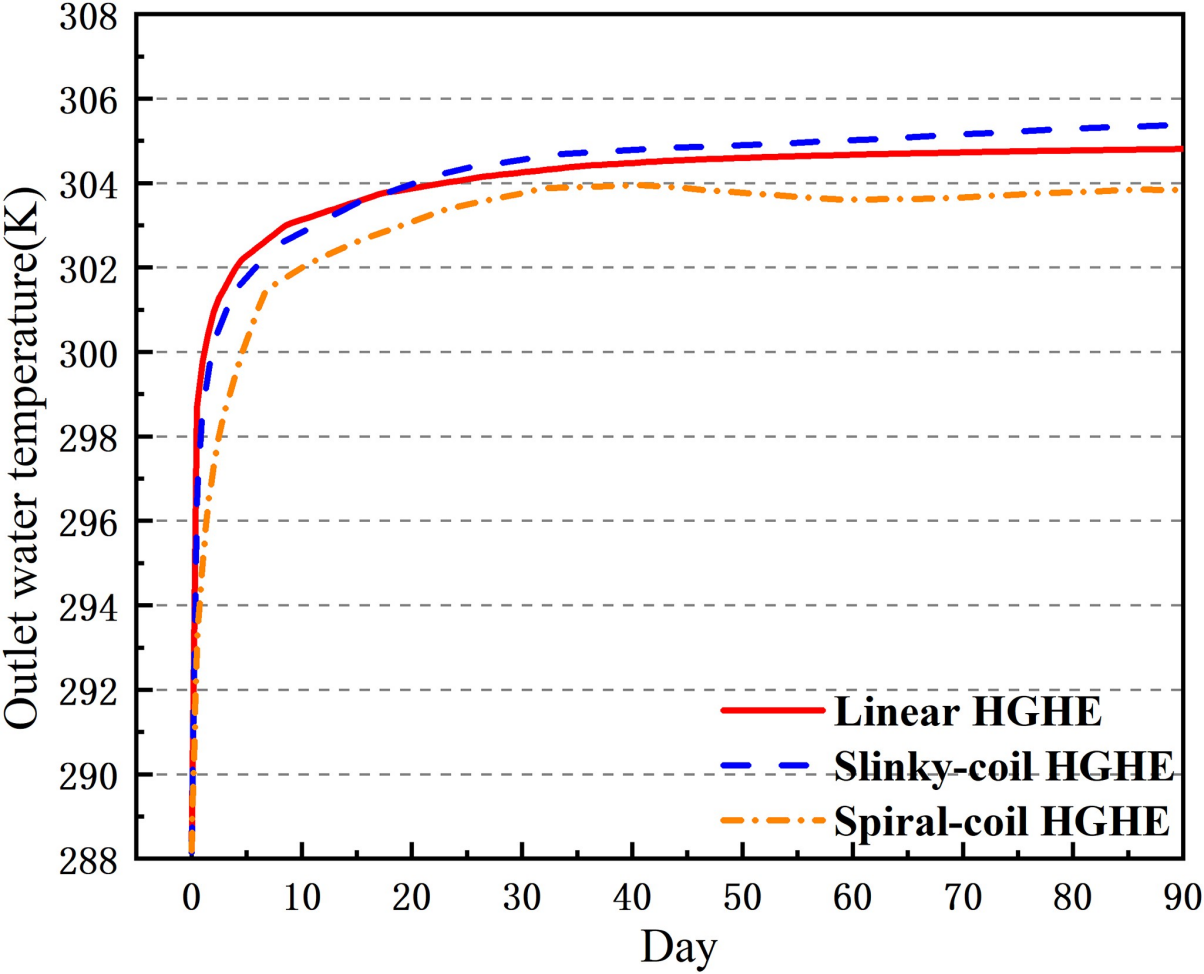
Outlet temperature of working fluid of HGHEs.

The outlet temperature of HGHEs is an important parameter used when evaluating the heat transfer performance of an underground system: a low difference between inlet and outlet working fluid temperatures implies poor heat transfer performance of ground heat exchangers with soils. As shown in Fig 12, the outlet water temperature of spiral-coil HGHEs is 303.8 K, showing a difference of 4.3 K from the inlet water temperature. This indicates that the exchangers present a strong heat transfer performance with surrounding soils, thus leading to a significant cooling effect.

Aiming at slinky-coil and linear HGHEs, its outlet temperature is slightly lower than that of linear HGHEs in the early stage of the cooling season. The reason for this is that the total laying length of slinky-coil HGHEs is large and the contact, and hence the working fluid traveling length and heat transfer area between the exchangers and surrounding soils is large, therefore, the heat transfer of slinky-coil HGHEs is stronger than the linear one. However, as heat is constantly input, the temperature of the surrounding soils increases.

As the spacing of slinky-coil HGHEs is narrow and thermal interference occurs between heat exchangers, the heat transfer performance of the slinky-coil HGHEs with the surrounding soils decreases in the later stage.

### 5.5 COP of units

The COP for reflecting the cooling performance of a heat pump refers to the ratio of the cooling capacity to the input power of units. As the COP linearly varies with the outlet temperature of heat exchanger [30–33], it is feasible to express the COP with the simplified alternative equation rather than directly employing the outlet temperature to evaluate their performance. Based on the test data of the SI 30TER+ units provided by the manufacturer (Dimplex), the following relationship is derived [30]:

$$COP_{c}=\alpha T_{out}+\beta$$ (22)

where, *T*_*out*_ refers to the outlet water temperature (°C) of a heat exchanger;*α* and *β* denote the coefficients (separately, -0.12 and 8.6 during cooling).

**Fig 13:**
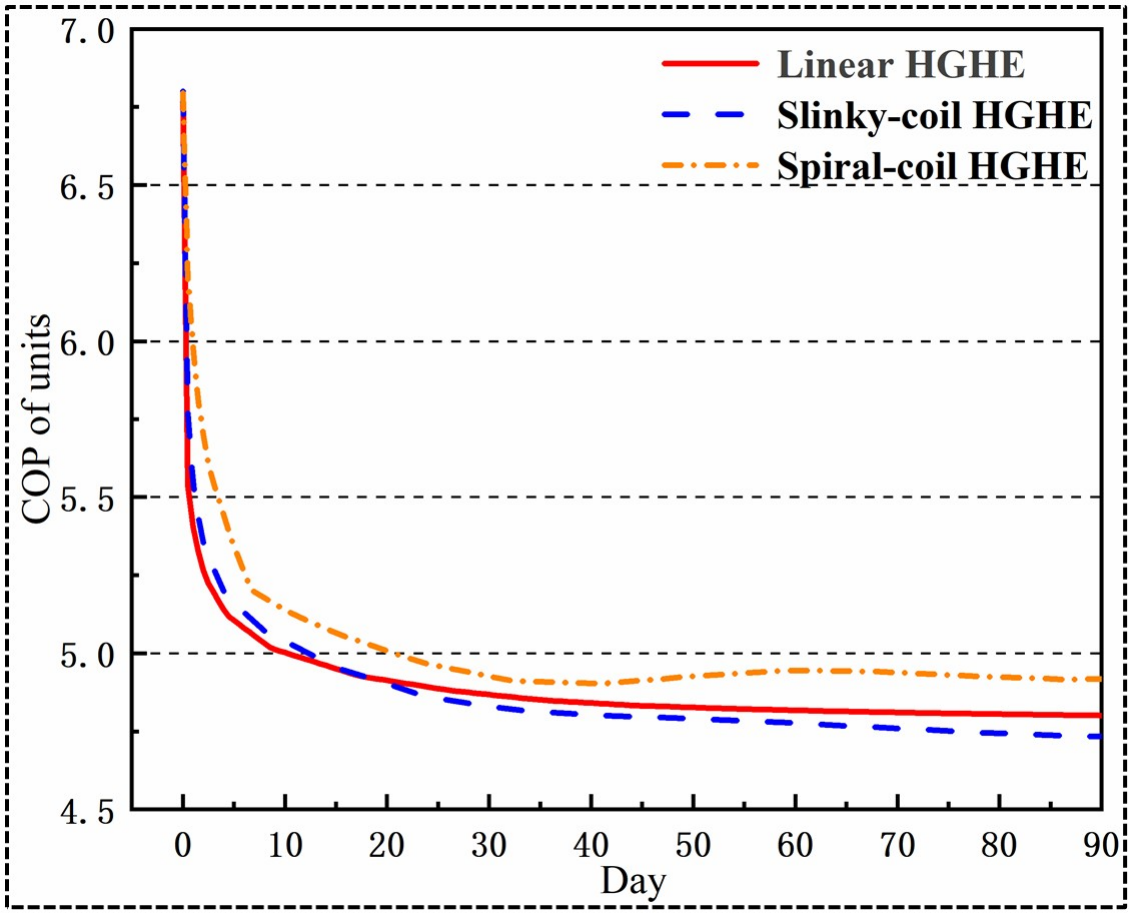
Outlet temperature of ground heat exchangers in the cooling stage.

Through analysis of the data in Fig 13, it can be found that the COP of the spiral-coil HGHEs throughout the cooling seasons is the highest, implying that the obtained economic benefit is large. In contrast, due to heat accumulation, slinky-coil HGHEs present a relatively poor heat transfer performance, showing a low COP, and its total heat transfer quantity of linear HGHEs is low due to being limited by the laying length. Therefore, it is not recommended to apply slinky-coil and linear HGHEs where the available space is limited.

### 5.6 Surplus temperature of energy-storage soils

A heat pump transfers heat with surrounding soils through ground heat exchangers. The cooling and heating are realised by inputting/absorbing heat into/from soils. However, the soils have a finite capacity for bearing heat and cold energy. Excessively inputting heat will lead to the constant growth of the soil temperature and further trigger heat accumulation. Similarly, excessively absorbing heat from soils induces the cold accumulation. The two problems are called heat imbalance and unfavourable to the long-term operation of a heat pump system.

**Fig 14:**
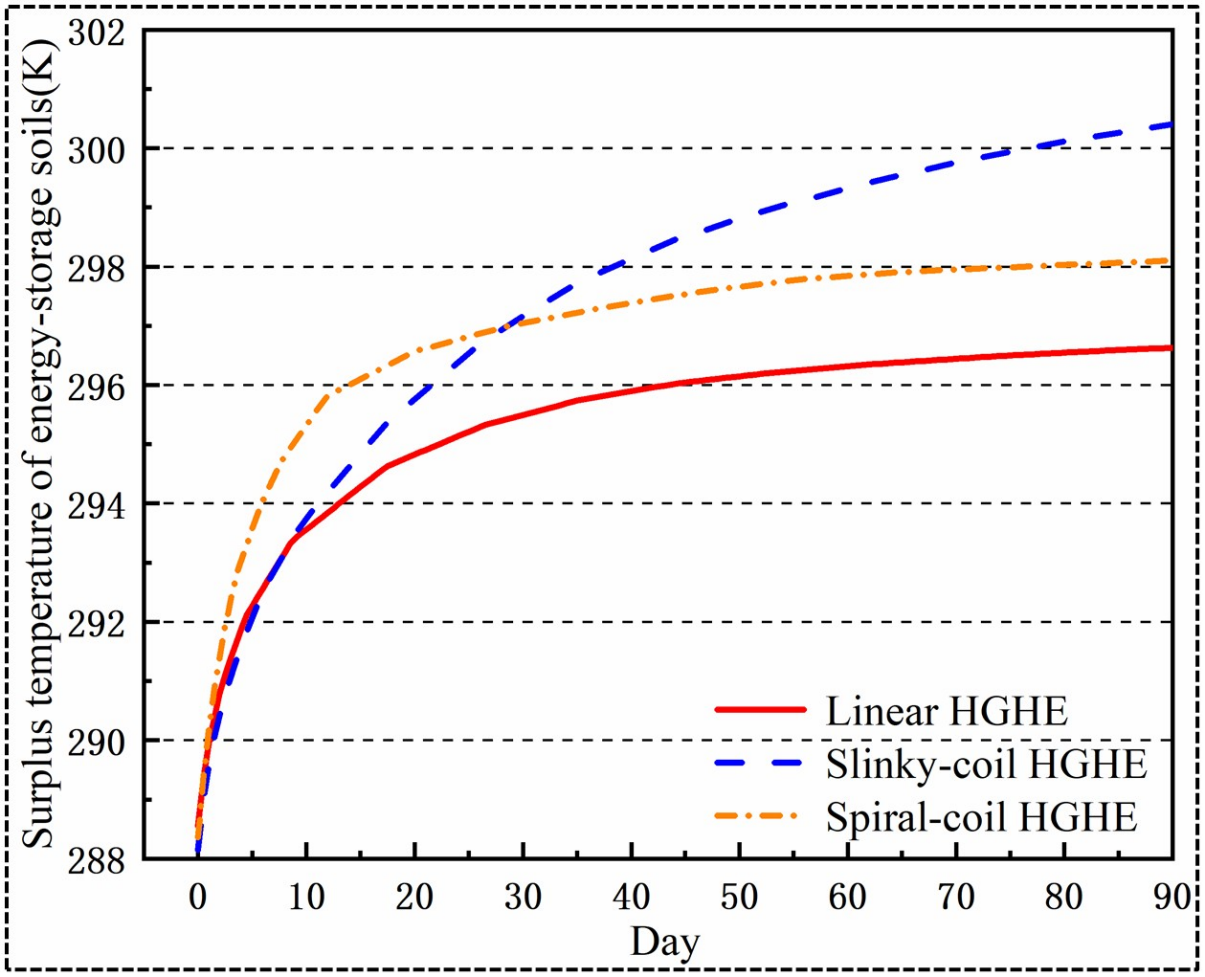
Surplus temperature of energy-storage soils.

As shown in Fig 14, the initial average temperature of energy-storage soils is 288.15 K. As heat is constantly input into soils by HGHEs, the temperatures of energy-storage soils all gradually rise. Data suggest that the average temperatures of energy-storage soils when applying the linear, spiral-coil and slinky-coil HGHEs separately increase to 296.6 K, 298.1 K, and 300.4 K after 90 days of cooling season operation. The temperature increase of energy-storage soils when applying slinky-coil HGHEs is 45% higher than that when employing linear HGHEs, which further reduces the temperature difference between the energy-storage soils and the inlet of HGHEs, thus, it is unfavourable for the heat transfer capacity of HGHEs.

In practices, construction cost and maintenance should be considered when compare different options of HGHEs. Since the price of PE tube usually is low, variation in tube length unlikely leads to significant change of construction cost. Besides, the ditch sizes in three types of HGHEs are the same in this study, hence the excavation cost is similar. To summarize, the construction cost for these three types of HGHEs varies little. Few maintenance is required once it is finished. Since HGHEs are buried shallowly, pumping power to circulate working fluid is minimal and very similar for these three types.

## 6 Conclusion

In this study, the long-term heat transfer performance of liner, spiral-coil and slinky-coil HGHEs were simulated and investigated. Compared with the traditional linear HGHEs, the spiral-coil and slinky-coil HGHEs save on the use of significant site area; longer heat exchangers can be arranged per area, contributing to a high land utilisation ratio; as well as a large heat transfer area.

Under cooling conditions, it is feasible to attain a lower outlet working fluid temperature by applying spiral-coil HGHEs. Simulation results have shown that the outlet temperatures of spiral-coil, linear and slinky-coil HGHEs are 303.8 K, 304.8 K, and 305.4 K, respectively. From the perspective of the heat transfer performance, the spiral-coil HGHEs present the best heat transfer performance, with a higher COP of units. Thus, within a limited site area, it is possible to attain higher benefit by using spiral-coil HGHEs.

Heat accumulation, or heat imbalance in a more general sense, is one of the major problems associated with GCHP operation. In this study, after cooling for 90 d with linear HGHEs, the increase in the temperature of energy-storage soils is relatively small, suggesting that the risk of heat imbalance is low. The linear HGHEs show a high heat transfer efficiency; however, one has to remind that the total capacity of linear HGHEs is the lowest. The temperature of energy-storage soils increases significantly when applying slinky-coil HGHEs, which induces a low heat transfer per linear metre of HGHEs, thus showing a low COP. Considering the economics and user demand for total cooling capacity, it is advised to employ spiral-coil HGHEs.

It is inevitable that some thermal interference is generated between the working fluid-supply and return sides, which affects the heat transfer performance of the HGHEs. When applying linear HGHEs, the extent of this thermal interference between heat exchangers is the lowest; that between slinky-coil HGHEs is the highest. Thus, with a fixed trench width for the HGHEs, it is not advised to apply slinky-coil HGHEs.

The parameters of heat-affected area and heat transfer capacity per heat-affected area are first proposed in this study to better assess the land utilization efficiency and economics of different types of HGHEs in practice. The simulation results demonstrate that the spiral-coil HGHEs has the highest value of heat transfer capacity per unit area. The concept provides a new tool to evaluate the advantages of various HGHEs.

## Abbreviations

*(hZ)_eff_*: *Effective value of the thermal conductivity*
*(ρC_p_)_eff_*: *Effective volumetric heat capacity*, *J/kg∙K*
*▽ψ*: *Difference of water potentials at any two points*
*A*: *Cross-sectional area of the fluid flow*, *m*^*2*^
*a,n,m*: *Scale paramter*
*C_p_*: *Specific heat capacity at constant pressure*, *J/kg∙K*
*C_p,f_*: *Specific heat capacity at constant pressure of fluids in heat exchanger tubes*, *J/kg∙K*
*d_h_*: *Hydraulic mean diameter*,*mm*
*f_D_*: *Darcy’s friction coefficient*
*h*: *Equivalent convection heat transfer coefficient of the pipe wall*, *W/m*^*2*^*∙K*
*h*: *Negative pressure*, *cmH*_*2*_*O*
*k(θ)*: *Permeability coefficient which varies with the water volume fraction*
*k*: *Coefficient of thermal conductivity*, *W/m∙K*
*k_eff_*: *Effective thermal conductivity*, *W/m∙K*
*k_f_*: *Coefficient of thermal conductivity of circulated fluids*, *W/m∙K*
*Q_wall_*: *Heat transfer between the heat exchanger tube and their ambient environment*, *J*
*T*: *Temperature*, *K*
*t*: *Time*, *s*
*T_ext_*: *Temperature of the porous media outside the pipe wall*, *°C*
*T_f_*: *Temperature of circulated fluids*, *K*
*u*: *Velocity of the circulated fluids in ground heat exchangers*, *m/s*
*v*: *Darcy’s velocity*, *m/s*
*Z*: *Perimeter of the pipe wall*, *m*
*α, β*: *Coefficients*
*η*: *Soil porosity*
*θ,χ_w_*: *Volumetric water content*, *cm*^*3*^*/cm*^*3*^
*θr*: *Retention rate of water*, *cm*^*3*^*/cm*^*3*^
*θs*: *Saturated water content of water*, *cm*^*3*^*/cm*^*3*^
*κ*: *The permeability coefficient of porous media*, *m*^*2*^
*μ*: *Dynamic viscosity*, *Pa∙s*
*ρ*: *Density of porous media*, *kg/m*^*3*^
*ρ_f_*: *Fluid density*, *kg/m*^*3*^
*ρ_w_*: *Density of groundwater*, *kg/m*^*3*^
*χ_ai_*: *Volumetric air content*, *cm*^*3*^*/cm*^*3*^
*χ_s_*: *Volumetric soil content*, *cm*^*3*^*/cm*^*3*^
*ψ*: *Total soil water potential*
*ψ_g_*: *Gravity potential*
*ψ_m_*: *Solute potential*
*ψ_p_*: *Pressure potential*
*ψ_θ_*: *Matric potential*
